# Venous thromboembolism after oral and maxillofacial oncologic surgery: Report and analysis of 14 cases in Chinese population

**DOI:** 10.4317/medoral.21399

**Published:** 2016-12-06

**Authors:** Yang Wang, Jiannan Liu, Xuelai Yin, Jingzhou Hu, Evagelos Kalfarentzos, Chenping Zhang, Liqun Xu

**Affiliations:** 1 DDS, PHD, Resident doctor. Department of Oral maxillofacial-Head and Neck Oncology, Shanghai Ninth People’s Hospital, College of Stomatology, Shanghai Jiao Tong University School of Medicine, Shanghai Key Laboratory of Stomatology and Shanghai Research Institute of Stomatology, Shanghai, China; 2 DDS, PHD, Professor. Department of Oral maxillofacial-Head and Neck Oncology, Shanghai Ninth People’s Hospital, College of Stomatology, Shanghai Jiao Tong University School of Medicine, Shanghai Key Laboratory of Stomatology and Shanghai Research Institute of Stomatology, Shanghai, China; 3 MD, DDS, PHD, Resident doctor. Department of oral & maxillofacial surgery, University of Athens dental school; 4 DDS, PHD, Professor. Department Head, Department of Oral maxillofacial-Head and Neck Oncology, Shanghai Ninth People’s Hospital, College of Stomatology, Shanghai Jiao Tong University School of Medicine, Shanghai Key Laboratory of Stomatology and Shanghai Research Institute of Stomatology, Shanghai, China

## Abstract

**Background:**

Venous thromboembolism (VTE) including deep vein thrombosis (DVT) and pulmonary embolism (PE) is a leading cause of death in cancer patients. The aim of this study was to explore the potential risk factor of VTE in oral and maxillofacial oncological surgery.

**Material and Methods:**

The data of patients who received operation in our institution were gathered in this retrospective study. A diagnosis of VTE was screened and confirmed by computer tomography angiography (CTA) of pulmonary artery or ultrasonography examination of lower extremity. Medical history and all perioperative details were analyzed.

**Results:**

14 patients were diagnosed as VTE, including 6 cases of PE, 7 cases of DVT, 1case of DVT and PE. The mean age of these patients was 62.07 years. Reconstruction was performed in 12 patients of these cases, most of which were diagnosed as malignance. Mean length of surgery was 8.74 hours, and lower extremity deep venous cannula (DVC) was performed in all these patients.

**Conclusions:**

We analyzed several characters of oral and maxillofacial surgery and suggested pay attention to lower extremity DVC which had a high correlation with DVT according to our data.

**Key words:**Venous thromboembolism, pulmonary embolism, deep vein thrombosis, oral and maxillofacial surgery.

## Introduction

Thromboembolism is a well recognized complication of malignant disease and it is known that malignant disease itself can predispose to a hyper-coagulable state secondary to polycythemia vera. And cancer patients have a higher incidence of venous thromboembolism (VTE), including pulmonary embolism (PE) and deep venous thrombosis (DVT), compared to the general population (1). Therefore, cancer patients undergoing major surgery are considered particularly high risk for VTE.

The incidence of thromboembolic disease in general population is relatively low, about 0.1%-0.3% per year while among cancer patients, the occurrence of VTE is 4–7 times higher, depending on the type and the stage of cancer (2). Several retrospective studies revealed the incidence of VTE in head and neck cancer patients between 0.1% and 2% (3-7). The largest review was published by Moreano (3) and reviewed the records of 12,805 otolaryngologic patients with an overall incidence of DVT and pulmonary embolism of 0.3% and 0.2%, respectively. Patients with head and neck cancer usually undergo comprehensive resection and reconstruction and frequently exhibit many of the major risk factors for DVT and PE. These risk factors include advanced age, postoperative immobility, prolonged surgical procedure and *et al.* In accord with current criteria, most patients with head and neck cancer who have surgery are presumed to be high risk for developing VTE, especially for patients undergoing simultaneous reconstruction. The purpose of this study was to identify potential risk factors of VTE in oral and maxillofacial oncological surgery.

## Material and Methods

This study was approved by the Ethics Committee of Shanghai Ninth People’s Hospital. All the patients with tumor, including benign or malignant tumors, discharged from January 2011 to December 2015, that received operation in Oral Maxillofacial-Head Neck Department, Shanghai 9th Hospital were selected for study. All the detail of medical history was recorded, including gender, age, smoke/alcohol history, comorbidities, previous surgeries, prior chemotherapy or radiation therapy, prior VTE history, histology, preoperative D-dimer and *et al.* The characters of the surgery procedure were recorded including operative time, blood loss, blood transfusion, lower extremity DVC, simultaneous reconstruction and *et al.* Perioperative findings were summarized including abnormal symptoms and the time point as discovered, diagnosis method, treatment and other complications ( Table 1- Table 3).

**Table 1:**
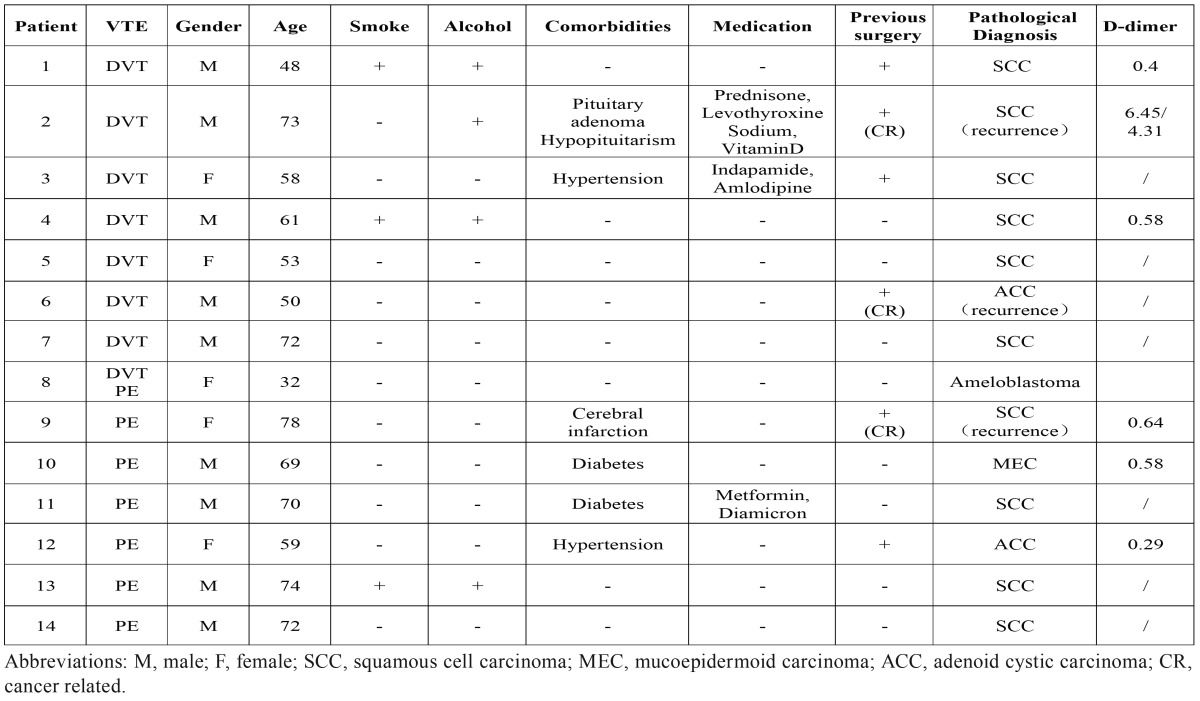
Preoperative medical record detail.

**Table 2:**
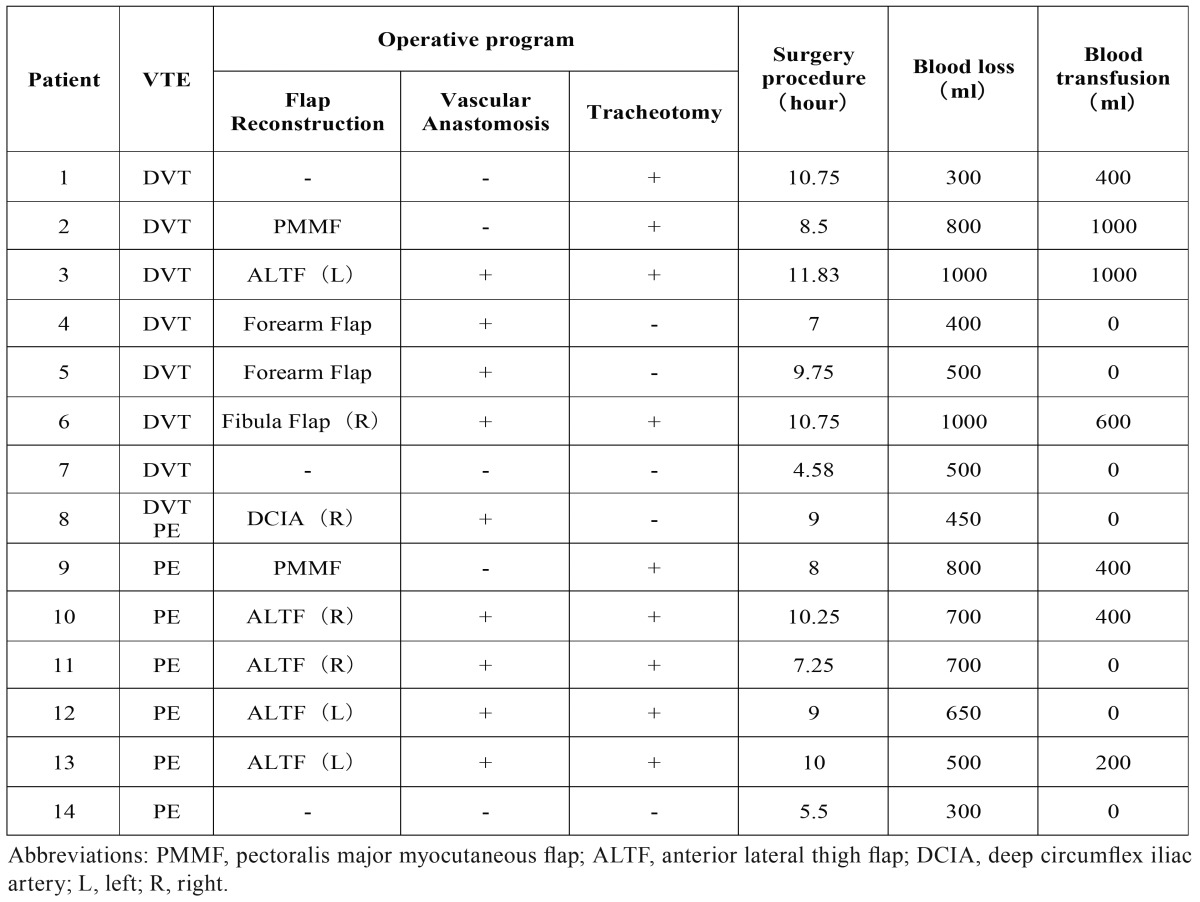
Medical record details of operation.

**Table 3:**
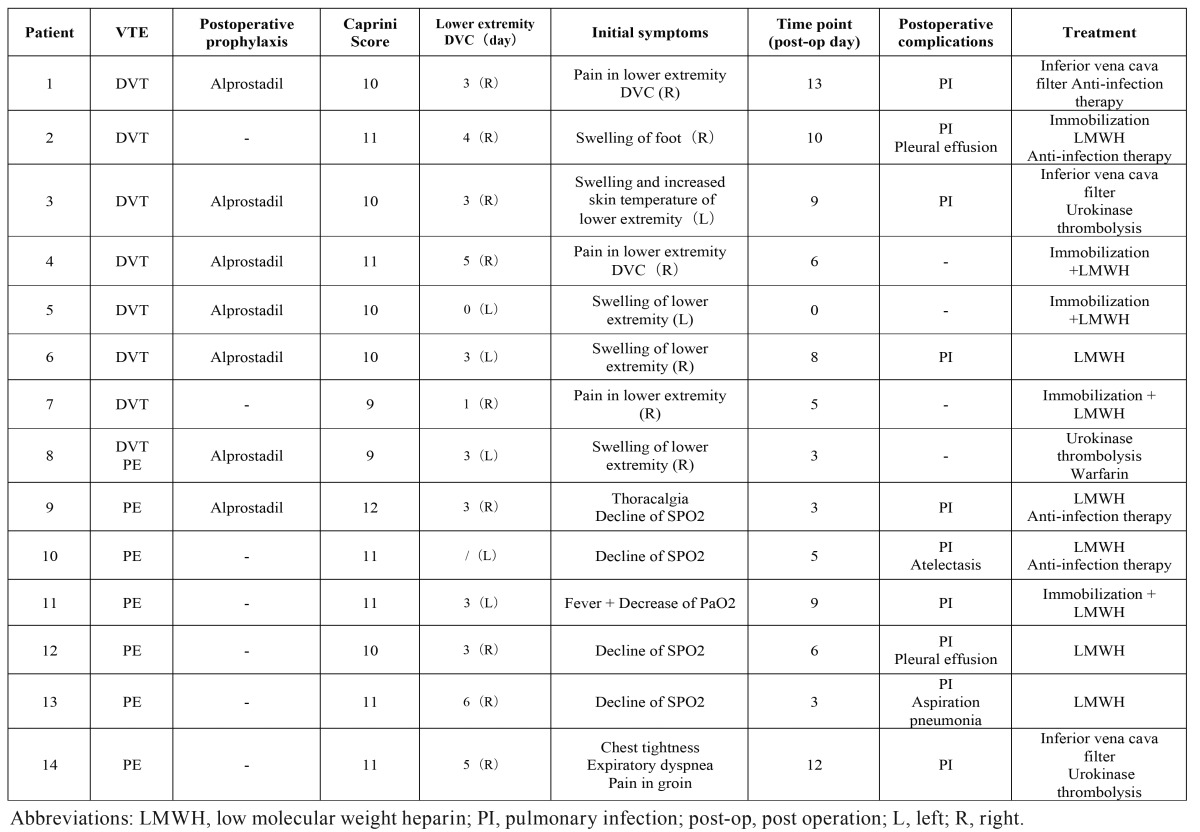
Postoperative medical record details.

Finally, a total of 9724 patients were included in this study and 14 patients were diagnosed as VTE, including 6 cases of PE, 7 cases of DVT, 1case of DVT and PE. The overall incidence of VTE was about 0.14%. The diagnoses were confirmed by computer tomography angiography (CTA) of pulmonary artery or ultrasonography examination of lower extremity.

## Results

The mean age of these patients was 62.07 years (±12.95; range from 32 to 78). There were 9 men and 5 women. 4 patients had a smoking history and 5 patients had a history of alcohol intake. In the term of basic disease, 2 patients had a history of hypertension and 2 patients had got diabetes. 6 patients had suffered a previous surgery including 3 malignance resulted in operation, and 1 operation was lower extremity involved. One patient had received a prior chemotherapy and radiotherapy ( Table 4). Reconstruction was performed in 12 patients of these cases, most of which were all diagnosed as malignance, including 1 fibula flap, 1 DCIA flap, 2 forearm flaps, 2 pectoris major myocutenous flaps (PMMF) and 6 anterior lateral thigh flaps (ALTF) ( Table 5).

**Table 4:**
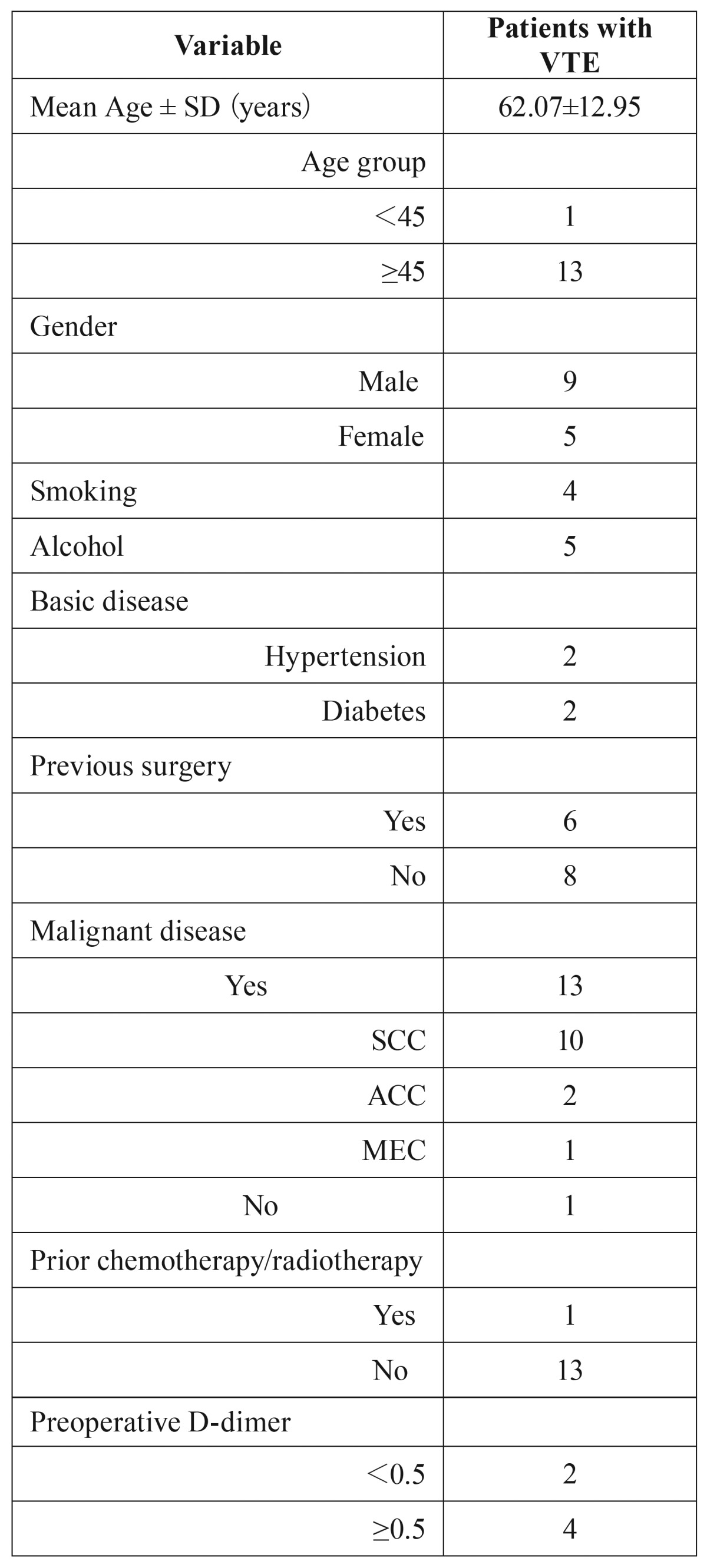
Patient characteristics.

**Table 5:**
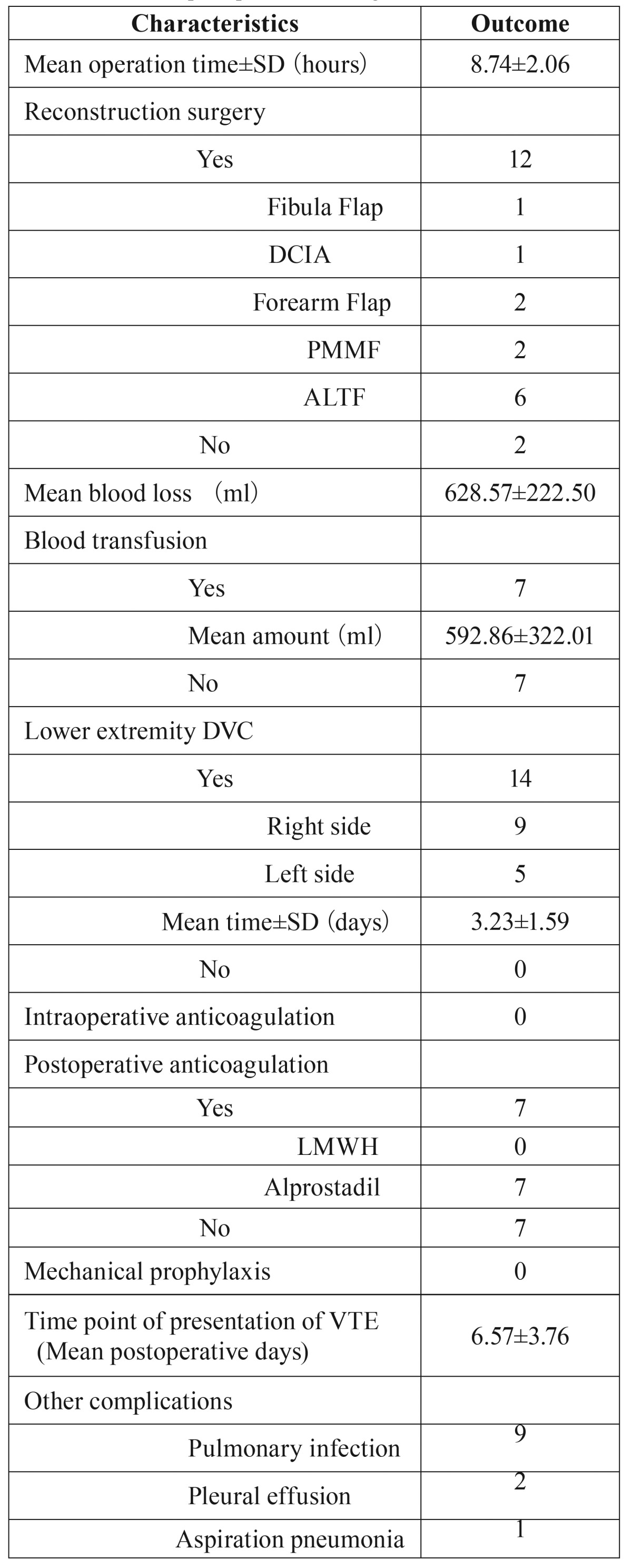
Statistical perioperative findings.

Mean length of surgery was 8.74 hours (±2.06; range from 4.58 to 10.75). Mean blood loss was 628.57ml (± 222.50; range from 1000 to 300), and 7 patients got blood transfusion and mean amount was 592.86ml(±322.01; range from 200 to 1000) ( Table 5). Lower extremity DVC was performed in all 14 patients via femoral vein puncture, 5 cases in left side and 9 cases in right side. Among the 8 patients of DVT, 5 cases occurred on the ipsilateral of DVC and initial symptom was the pain and swelling at the position of DVC. The other 3 cases occurred on the ipsilateral of flap and initial symptom was the pain and swelling of the lower extremity ( Table 3). The mean time of DVC usage was 3.23 days (±1.59; range from 0 to 6) ( Table 5). DVC was usually removed within 5 days, and the rule was broken only in 1 patient whose DVC was removed in the 6th day. In particular, one patient was discovered swelling in the position of femoral vein puncture in the end of the surgery and a diagnosis of DVT was made. For this case, the time of DVC usage was recorded as 0 day ( Table 3).

**Figure 1:**
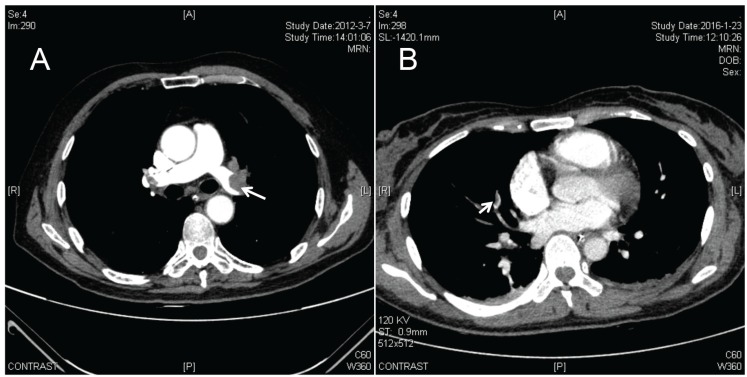
PE diagnosed by CTA of pulmonary artery. (A) Pulmonary artery trunk was embolized. (B) Pulmonary arteriole was embolized. The arrows show the thrombus.

Postoperative prophylaxis was recorded. Anticoagulation was performed in 7 patients and alprostadil was used rather than LMWH (low molecular weight heparin) ( Table 3). Mechanical prophylaxis, neither graduated elastic stocking (GES) nor intermittent pneumatic compression (IHC) was performed. The time point of presentation of VTE ranged from the end of the surgery to 13th day post operation, and the mean was 6.57 days (±3.76) post operation ( Table 5). Most of the initial symptom of DVT was pain and swelling of lower extremity whereas decline of oxygen saturation (SPO2) and pressure of arterial oxygen (PaO2) was the most common initial symptom of PE ( Table 3). For the cases of PE, pulmonary artery trunk was embolized in one case (Fig. 1A), and inferior vena cava filter and urokinase thrombolysis was performed. For the cases whose pulmonary arteriole was embolized (Fig. 1B), pulmonary infection was always discovered; the decline of SPO2 was corrected when LMWH and anti-infection therapy were performed simultaneously. For the cases of DVT, immobilization and LMWH were required in every case; inferior vena cava filter was employed in 2 cases in case of deadly PE ( Table 3).

## Discussion

The pathophysiology of VTE was elucidated by Virchow more than 150 years ago and Virchow’s triad, comprising three elements - venous stasis, endothelial injury, and hypercoagulability - is still used to explain its development (8). In a review, Anderson and Spencer (8) verified that active cancer, together with advanced age, prolonged immobilization, type of operation, serious injury, previous VTE, and congestive heart failure, are “convincingly demonstrated” independent risk factors. Obesity, use of nicotine, chemotherapy, red cell transfusion, or coexisting medical conditions, could further increase the risk.

The incidence of thromboembolic disease in general population is relatively low. Malignant diseases are considered a serious risk factor for the development of VTE (9). The incidence of VTE in head and neck cancer patients was reported as between 0.1% and 2%. However, it should be kept in mind that the true rates could have been underestimated because of the limitations of these studies. First, not all patients with VTE become symptomatic. Second, the analysis relied, like all retrospective studies, on adequate record keeping. So far, rare had been reported in Chinese population on the topic of VTE, especially in oral and maxillofacial surgery.

In our study, several special characters had been revealed in oral and maxillofacial cancer patients and the perioperative treatment.

First, patients who suffered oral and maxillofacial cancer were usually elder. In our study of these 14 cases, 13 patients were over 45 years old, 9 patients over 60 and 6 patients over 70. The mean age was 62.07 years ( Table 1, Table 4). And age was found to be a risk factor in patients with cancer associated VTE in several studies. On the other hand, occurrence of systemic disorder, for example hypertension, diabetes and cerebral infraction, in elder patients was higher than younger patient, which make the risk of VTE higher.

Second, oral and maxillofacial cancer patients usually need a radical resection, and the simultaneous reconstruction is necessary for functional and aesthetical requirement. Flap transplantation for the reconstruction of recipient defect always prolonged the surgery procedure, otherwise, for the sake of adequate blood supply to the flap in head and neck region, and avoiding twist of vascular pedicel, patients with flap transplantation were required to lie on the back and immobilize at least for 3 days according to our department’s standard of care. In spite of lower extremity massage, immobilization increased the risk of VTE.

In our study, reconstruction was performed in 12 patients of these cases including 10 vascularized free flaps and 2 pedicel flaps ( Table 5). ALTF and fibula flap were now very popular for reconstruction of soft tissue and osseous tissue defect respectively. In our study, three cases of DVT occurred on the ipsilateral of flap elevation, including ALTF, fibula flap and DCIA. 4 in 6 cases of PE got a reconstruction with ALTF. During flap elevation, especially flaps involving lower extremity, GES and IHC were not convenient to use. Also, the transection and division make injury to lower extremity vessel, which was potential risk factor of DVT.

Third, DVC was applied universally during major surgery in our department. Among the 8 patients of DVT, 5 cases occurred on the ipsilateral of DVC and initial symptom was the pain and swelling at the puncture position ( Table 3). The most favorite puncture position of DVC was right femoral vein because of increased potential risk of thrombosis in left femoral vein, which was oppressed by iliac artery anatomically. Also, the puncture position verified depending on the donor site of flaps. Anyway, lower extremity DVC has a high correlation with DVT according to our study. Although central venous access was recognized as a risk factor for VTE, rare had reported the correlation between DVT and lower extremity DVC as in our study.

Additionally, neck dissection was frequently necessary in head and neck cancer surgery because of high rate of lymph node metastasis. The division of jugular vein may result in endothelial injury, which was consistent with Virchow’s triad. Further more, the upper respiratory tract was usually involved in head and neck cancer surgery. To alleviate swelling and get airway unobstructed as soon as possible, high-dose glucocorticoid, which was elucidated as a risk factor of VTE, was applied.

So far, more than 25 evidence-based guidelines on the prevention of VTE by the appropriate use of prophylaxis have been developed on the basis of results of clinical trials in different cohorts of at-risk patients since 1986. These include guidelines published by the American College of Chest Physicians (ACCP) (10-12), the ninth edition of which was published in 2012 and those issued by the American Association of Orthopedic Surgeons (AAOS) (13) in 2012, which offer substantial insight into the risk of VTE as well as appropriate prophylaxis measures in orthopedic patients. Unfortunately there was no guideline for oral and maxillofacial surgery specifically.

Understanding guidelines and recommendations from other specialties will help us to make more appropriate decision for our patients. The most widely used risk assessment tool was developed by Caprini (14). The Caprini risk assessment tool has been updated periodically since 30 years ago. According to this model, approximately 40 risk factors are listed, with weights of 1 to 5 points each. The total risk factor score is then used to group patients into 1 of 4 categories (low, moderate, high, and highest risk), each with a recommended prophylactic treatment. The Caprini risk assessment tool has been validated in retrospective studies for use in patients needing elective general surgery and modified for use in patients needing plastic surgery (15-18). We make a Caprini score for every case according to ACCP9 retrospectively, found that every patient got a very high Caprini score (score≥9) ( Table 3) and fell into high-risk group which should be dealt with aggressive thromboprophylaxis. But in reality, only prophylactic alprostadil was used in 7 cases, no postoperative LMWH was applied prophylactically. The main concern was that routine prophylactic anticoagulation will increase the risk of postoperative hematoma, which can compromise flap viability or upper respiratory tract. Thus, currently few oral and maxillofacial surgeons routinely prescribe anticoagulation after resection and microvascular reconstruction. Meta-analysis has shown that pneumatic compression devices also decrease DVT in patients undergoing surgical procedures by over 60%. But unfortunately it was not available in our department. GES was also not widespread generalized because of apathetic awareness of VTE.

## Conclusions

In this study we aimed to identify potential risk factors for developing VTE for patients with head and neck cancer. We analyzed several characters of oral and maxillofacial surgery and suggested pay attention to lower extremity DVC which had a high correlation with DVT according to our data. And challenges to patient care in our department include lack of equipment like IHC, application of GES and *et al.*
